# Review of "Evolution in Four Dimensions: Genetic, Epigenetic, Behavioral, and Symbolic Variation in the History of Life" by Eva Jablonka and Marion J. Lamb

**DOI:** 10.1186/1475-925X-4-68

**Published:** 2005-12-21

**Authors:** Andrew Lloyd

**Affiliations:** 1 Education & Research Centre, St. Vincent's University Hospital, Dublin Ireland

Reductionism rules. Progress in science is made by simplifying the daunting complexity of the living world into a series of controlled experiments or observations. We use model organisms to help this simplification and eventually nail down some few facts on which everyone agrees. We agree that something is true for a particular gene at a particular time, in a particular population in a particular species. When we write the paper, however, we imply that this truth is much more widely applicable genetically, temporally and taxonomically. When pushed, all the biological scientists I know would admit to "believing in evolution" but few have read the Origin of Species, and very few could elaborate the evidence on which this belief is based. They cannot, after all, critically evaluate the data from molecular biology, geology, genetics, biogeography, ethology, biochemistry, mathematics and ecology in one lifetime. If diseases cannot be explained by a simple Mendelian model (and only 2% of entries in OMIM can), it is not necessarily always fruitful to invoke a more complex multigene, but still essentially Mendelian, model for the other 98%.

Jablonka and Lamb have written a tendentious (exercising affirmative action on behalf of minority/iconoclastic ideas) book to urge us to look at the assumptions we make when trying to make sense of evolution. In particular they look with gimlet eyes at the Neo-Darwinian orthodoxy, which asserts that evolution occurs when gene frequencies change and that this happens by population genetics quadrivium of mutation, migration, selection and drift.

They consider how evolution might occur in ways which by-pass the Central Dogma of DNA makes RNA; RNA makes protein; and proteins make everything else. In particular they fish for cases of Lamarckian evolution where the events of one lifetime change the phenotype and can somehow be heritably transmitted to succeeding generations. They find a number of fascinating facets of biology which deserve an explanation and which appear to be poorly served by regular gene-based evolutionary theory.

They work through each case (and each case is chosen from the whole of Pubmed to best illustrate a particular phenomenon) to establish whether it is feasible and give what evidence they can that it is likely. But it is still a long way from here to establish whether these examples are interesting warts (well, beauty-spots, then) on the face of Neo-Darwinian understanding or a tumour which if scratched will expose current dogma as an empty or deeply flawed shell.

The book is engagingly well-written, and illustrated with quirky cartoons by Anna Zeligowsky. Following a well-established philosophical tradition, each chapter is wrapped up by a dialogue between the authors and a character representing the Alternative View. They have not repeated Galileo's near-fatal alienating error of dubbing the foil "Simplicius" and these dialogues fairly put forward some possible objections from orthodoxy and common sense. Thirty pages of Notes, which are an interesting read in themselves, are followed by a comprehensive bibliography and a competent index. If you're happy with your Neo-Darwinian certainties, you'll find much to bristle at in this book. If you think we are due for a paradigm shift in evolutionary biology, then you can certainly catch the breeze of it here.

